# Application of layered poly (L-lactic acid) cell free scaffold in a rabbit rotator cuff defect model

**DOI:** 10.1186/1758-2555-3-29

**Published:** 2011-12-02

**Authors:** Atsuyuki Inui, Takeshi Kokubu, Hiroyuki Fujioka, Issei Nagura, Ryosuke Sakata, Hanako Nishimoto, Masaru Kotera, Takashi Nishino, Masahiro Kurosaka

**Affiliations:** 1 Department of Orthopaedic Surgery, Kobe University Graduate School of Medicine 7-5-1 Kusunoki-cho, Chuo-ku, Kobe, 650-0017, Japan; 2 School of Rehabilitation, Hyogo University of Health Sciences, 1-3-6 Minatojima, Chuo-ku, Kobe, 650-8530, Japan; 3 Department of Chemical Science and Engineering, Kobe University Graduate School of Engineering, 1-1 Rokkodai-Cho, Nada-ku, Kobe, 657-8501, Japan

## Abstract

**Background:**

This study evaluated the application of a layered cell free poly (L-lactic acid) (PLLA) scaffold to regenerate an infraspinatus tendon defect in a rabbit model. We hypothesized that PLLA scaffold without cultivated cells would lead to regeneration of tissue with mechanical properties similar to reattached infraspinatus without tendon defects.

**Methods:**

Layered PLLA fabric with a smooth surface on one side and a pile-finished surface on the other side was used. Novel form of layered PLLA scaffold was created by superimposing 2 PLLA fabrics. Defects of the infraspinatus tendon were created in 32 rabbits and the PLLA scaffolds were transplanted, four rabbits were used as normal control. Contralateral infraspinatus tendons were reattached to humeral head without scaffold implantation. Histological and mechanical evaluations were performed at 4, 8, and 16 weeks after operation.

**Results:**

At 4 weeks postoperatively, cell migration was observed in the interstice of the PLLA fibers. Regenerated tissue was directly connected to the bone composed mainly of type III collagen, at 16 weeks postoperatively. The ultimate failure load increased in a time-dependent manner and no statistical difference was seen between normal infraspinatus tendon and scaffold group at 8 and 16 weeks postoperatively. There were no differences between scaffold group and reattach group at each time of point. The stiffness did not improve significantly in both groups.

**Conclusions:**

A novel form of layered PLLA scaffold has the potential to induce cell migration into the scaffold and to bridge the tendon defect with mechanical properties similar to reattached infraspinatus tendon model.

## Background

Rotator cuff tear is a common problem that may cause chronic pain with functional disability. If intolerable pain or functional disability persists, surgical treatment can be considered. Excellent outcomes have been reported with arthroscopic rotator cuff repair in small or medium sized tears [1], but large or massive rotator cuff tears are challenging for surgeons [2]. Surgical procedures are available for repair of massive rotator cuff tears including musculotendinous transfer [3] and patch grafting using fascial tissue [4] or synthetic materials [5-8], but these techniques have disadvantages. Tendon transfer is not an anatomical reconstruction and patch grafting using fascial tissue requires sacrifice of normal tissue. Using extracellular matrix derived from animals such as porcine small intestinal submucosa can cause an immune reaction and carry the risk of zoonoses resulting from xenotransplantation [9]. Nonabsorbable synthetic materials have been used [10], but foreign body reaction and bone erosion has been reported several years after surgery [11].

In tendon regeneration filed, tissue engineering using scaffolds, cultured cells and growth factors has been showing great advantage [12]. In a controlled laboratory study on rabbit model, fibroblast-seeded scaffolds showed better results compared to the scaffold alone in rotator cuff regeneration [6]. However, these methods require 2-stage surgeries; a first surgery to harvest cells and a second to transplant them. Since 2-step surgeries are considered invasive, less invasive methods need to be developed. If the scaffold could induce cells from the surrounding tissues, pre-expansion of the cells for the transplantation would not be required. The authors assessed a double layered form of bioabsorbable poly (L-lactic acid) (PLLA) fabric which have smooth surface on one side and pile-finished surface on the other side, and reported its potency for regenerations of tendons and ligaments in a rabbit model [13]. The author hypothesized that PLLA scaffold without cultivated cells would lead to regeneration of tissue with mechanical properties similar to reattached infraspinatus without tendon defects. In the current study, application of the layered PLLA scaffold was evaluated for the regeneration of rotator cuff tears in a rabbit model.

## Methods

All the animal experiments were approved by the Animal Research Committee.

### Preparation of Layered PLLA Scaffold

**Figure 1:**
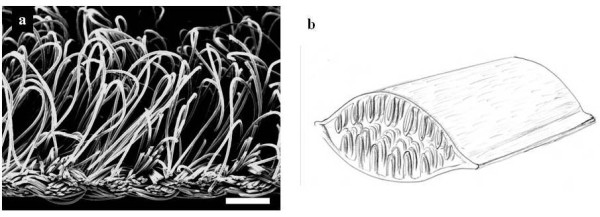
**Layered PLLA scaffold**. **(a) **SEM of the rough side of the pile-finished PLLA fabric. Scale bar represents 50 μm. **(b) **Schema of PLLA scaffold; two fabrics were superimposed in double layers with the smooth surface outside and rough surface inside, and both edges of the double layered fabric were bonded by thermocompression.

PLLA fibers (molecular weight, 86000; diameter, 23 μm) were textured into double layered fabric which had a smooth surface on one side and a rough (pile-finished) surface on the other side (Figure 1a). The fabric was cut to 5 × 10 mm pieces, two pieces were superimposed with the smooth surface outside and the rough surface inside. The both edges of the superimposed pieces were bonded by thermocompression. This tubular shaped fabric had layered structure, smooth outside and pile finished inside. Each tubular fabric was sterilized and used as layered scaffolds (Figure 1b).

### Scaffold Implantation

**Figure 2:**
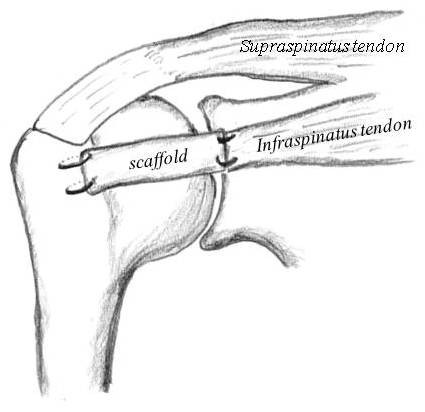
**A schematic drawing of the repair of the rotator cuff defect using PLLA scaffold in a rabbit model**. Defects in the infraspinatus tendons were created at their insertions and PLLA scaffolds were transplanted and both edges were fixed to tendon or bone by 4-0 nylon suture.

Thirty two female Japanese white rabbits (2.7-3.5 kg) were administered intravenous pentobarbital (30 mg/kg). The surgical area was disinfected and 3 ml of 1% lidocaine was injected subcutaneously. A 3 cm incision was made on the right shoulder and the infraspinatus tendon was exposed. Rotator cuff defects were created by resecting the infraspinatus tendon (5 mm in width and 5 mm in length) from the greater tuberosity, and the scaffolds were transplanted to repair this defect. The proximal edge of the scaffold was sutured to the midsubstance of the tendon by 4-0 nylon suture. Decortication was performed at the greater tuberosity of the humeral head (1 mm depth and 5 mm length) and the distal edge of the scaffold was fixed to the bone by 4-0 nylon suture (Figure 2). After repairing the defect, the muscle and skin was sutured in separate layers. In the contralateral shoulder infraspinatus tendon was resected without creating a defect and the edge of tendon was sutured directly to the humeral head by 4-0 nylon suture as a control. Rabbits were allowed free cage movement after the operation. Rabbits were euthanized with an intravenous fatal dose of sodium pentobarbital and infraspinatus tendon humeral head complex was excised. For macroscopic and histological evaluations, 4 rabbits were randomly selected at 4, 8, and 16 weeks after the operation. For mechanical analysis, 4 rabbits were randomly selected at 0, 4, 8, and 16 weeks after the operation. Four rabbits did not undergo surgery were examined mechanically as normal controls.

### Histological Analysis

After the macroscopic examination, the infraspinatus tendon humeral head complex was fixed in 4% paraformaldehyde, decalcified with 0.25 mol/l ethylenediaminetetraacetic acid in a phosphate buffered saline (pH 7.5), and embedded in paraffin. Sagittal sections (7 μm) were cut through the entire thickness of the defect, and stained with hematoxylin eosin (HE) then examined by light microscopy. For immunohistochemical analysis of type I and III collagens, deparaffinized sections were digested with proteinase (Dako Cytomation, Inc., CA, USA) for 10 minutes and treated with 3% hydrogen peroxide (WAKO Pure Chemical Industries, Ltd., Osaka, Japan) to block endogenous peroxidase activity. The sections were incubated with a 1:50 dilution of goat anti-type I or III collagen antibodies (Southern Biotechnology Associates Inc., AL, USA) at 4°C overnight and subsequently treated with peroxidase labeled anti-goat immunoglobulin (Histofine Simplestain max PO(G), Nichirei Bioscience, Tokyo, Japan) at room temperature for 30 minutes. The signal was developed using peroxidase substrate 3, 3-diaminobenzide (Histofine Simplestain DAB Solution, Nichirei Bioscience, Tokyo, Japan) and the sections were examined microscopically.

### Mechanical Analysis

The infraspinatus tendon humeral head complex was harvested from each shoulder and all soft tissue except for the infraspinatus tendon was resected by sharp dissection. The humerus was potted in specially designed devices using polymethylmethacrylate (PMMA) resin and a specially designed device clamped the end of each tendon. The complex was placed vertically to a tensile sensor (AG-I SHIMAZU Co, Kyoto, Japan). Before the tensile test was conducted, the infraspinatus tendon humeral head complex was preconditioned with a static preload of 0.5 N for 5 minutes, followed by 10 cycles of loading and unloading at strain amplitude of approximately 0.5% at a rate of 20 mm per minute. Immediately after preconditioning, the ultimate load to failure was recorded in uniaxial tension at 20 mm per minute [14]. The ultimate load to failure and the stiffness were measured from load-deformation curve. Mechanical properties of normal infraspinatus tendon and scaffold itself were evaluated in the same manner.

### Statistical Analysis

The paired *t *test was used to compare the mechanical properties of PLLA scaffold repair with the reattached tendon. Dunnett's test was used to compare the mechanical properties at each time point. A significance level of *p *< .05 was used. All data are presented as mean (SD).

## Results

### Histological Findings

**Figure 3:**
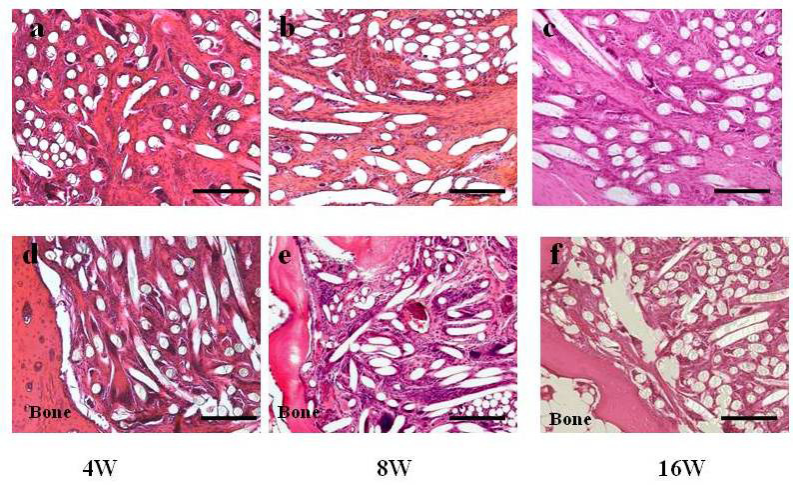
**HE staining of layered scaffold after implantation in rotator cuff defect in rabbit**. **(a) **scaffold midsubstance at 4 weeks after scaffold implantation. **(b) **scaffold midsubstance at 8 weeks after scaffold implantation. **(c) **scaffold midsubstance at 16 weeks after scaffold implantation. **(d) **scaffold bone interface at 4 weeks after implantation. **(e) **scaffold bone interface at 8 weeks after implantation. **(f) **scaffold bone interface at 16 weeks after implantation: Scale bar represents 100 μm.

**Figure 4:**
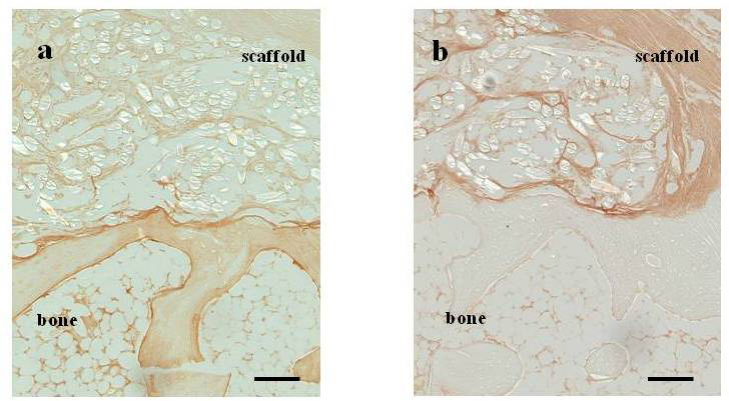
**Immunostaining of scaffold at 16 weeks postoperatively**. **(a) **type I collagen **(b) **type III collagen: type III collagen was mainly detected at the regenerated tissue in the scaffold. Scale bar represents 500 μm.

No gross evidence of infection was noted at the surgical site throughout the experiment, and all defects with the implanted layered PLLA scaffolds were covered with smooth fibrous tissue. At 4 weeks, after the operation, the scaffold persisted and spindle shaped cells were observed inside the pile-finished PLLA fibers (Figure 3a). At the interface of the scaffold and bone, which could be detected clearly, some fibrous tissue was observed inside of the scaffold (Figure 3d). At 8 weeks after the operation, spindle cells were observed inside the scaffold (Figure 3b), and regenerated tissue was directly connected to the bone with irregular alignment of the cells at the scaffold-bone interface (Figure 3e). At 16 weeks after operation, fibrous tissue like collagenous fibers were observed inside the scaffold and some of the fiber started to be absorbed (Figure 3c). Fibrous tissue connected to the bone at the tendon-bone interface, however chondrogenesis were not seen and a enthesis was not regenerated (Figure 3f). Some multinuclear cells were seen inside the scaffold at each time point. Type I collagen was not detected inside of the scaffold (Figure 4a), but type III collagen was mainly detected in regenerated tissue in the scaffold and fibrous tissue that covered the scaffold (Figure 4b).

### Mechanical Properties of Regenerated Tissue

**Figure 5:**
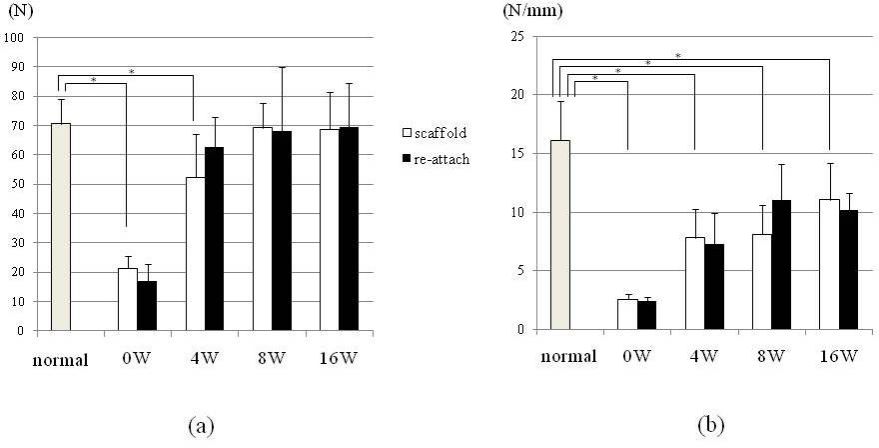
**Result of mechanical evaluation**. **(a) **Ultimate failure load of tendon humeral head complex. (Error bars indicate standard deviation) The ultimate failure load of scaffold group increased time dependently. It was significantly weaker at 4 weeks postoperatively than normal infraspinatus tendon; however statistical significant difference was not seen between 8 and 16 postoperative group and normal infraspinatus tendon. There was no significant difference between scaffold group and the reattach group at each time point. **(b) **Siffness of tendon humeral head complex. (Error bars indicate standard deviation) The stiffness of the scaffold group increased time dependently, however it remained significantly less stiff than that of normal infraspinatus tendon at each time point. Significant difference of stiffness was not seen between the scaffold group and the reattach group at each time point.

Mechanical examination revealed that the ultimate failure load of the scaffold group was 21.3 (4.2) N, 52 (14.7) N, 69 (8.4) N, and 68.8 (12.7) N at 0, 4, 8, and 16 weeks after the operation, respectively. The reattach group values were 16.9 (6) N, 61.7 (9.5) N, 78 (26.3) N, and 69.4 (14.9) N at 0, 4, 8, and 16 weeks after the operation, respectively. All samples were broken at the scaffold bone interface. The ultimate failure load of normal infraspinatus tendon was 70.6 (8.6) N and that of scaffold itself was 30.7 (6.2) N. The value obtained in the week immediately after the operation (week 0) mainly represents the mechanical property of the scaffold/suture construct. The ultimate failure load was weaker in the scaffold group at 4 weeks after the operation than normal infraspinatus tendon, but the ultimate failure load of scaffold group increased in a time-dependent manner and the difference between the scaffold group and normal infraspinatus tendon at 8 and 16 weeks was not statistically significant. In addition, there was no significant difference between the scaffold group and the reattach group at each time point (Figure 5a). On the other hand, the stiffness of the scaffold group was 2.6 (0.46) N/mm, 7.8 (2.5) N/mm, 8.1 (2.5) N/mm, and 11.1 (3.1) N/mm at 0, 4, 8, and 16 weeks after the operation, respectively. The reattach group values were 2.42 (0.32) N/mm, 7.3 (2.6) N/mm, 11.0 (3.1) N/mm, and 10.2 (1.4) N/mm at 0, 4, 8, and 16 weeks after the operation, respectively. The stiffness of normal infraspinatus tendon was 16.1 (3.4) N/mm and that of scaffold itself was 11.6 (4.2) N/mm. The stiffness of the scaffold group increased in a time-dependent manner, but it remained less stiff than the normal infraspinatus tendon at each time point. The stiffness was not significantly different between the scaffold group and the reattach group at each time point (Figure 5b).

## Discussion

Several synthetic materials have been previously used in rotator cuff regeneration. Implantation of polytetrafuluoroethylene (PTFE) patch grafts showed satisfactory functional results in 23 out of 25 patients with massive rotator cuff tears [10]. However, reconstruction of rotator cuff defects with PTFE in a canine model showed foreign body reaction at 12-24 weeks after surgery and bone resorption at the insertion of the PTFE into the bone [11]. Chitin fabric used as a scaffold in rabbit rotator cuff tears resulted in regenerated tissue, mainly composed by type III collagen [7]. Finally, poly glycolic acid (PGA) sheets used as a scaffold showed a well-arranged fibrocartilage layer in regenerated tendon insertions [8].

All the scaffolds previously used for rotator cuff tears had a uniform structure [5-8,10,11]. The authors previously reported the potency of double layered PLLA fabric which has smooth surface on one side and pile-finished surface on the other side for tendon tissue engineering. In its pile-finished side, cell migration was observed. On the other hand, smooth side showed less adhesion. The double layered fabric maintained its mechanical property compared with plain-woven fabric [13]. In present study, the authors chose this PLLA fabric as the scaffold material for rotator cuff repair study. A substantial amount of spindle shaped cells migrated inside the layered scaffold. The authors also noted several giant polynuclear cells indicative of an inflammatory reaction.

PLLA fiber is an organic polymer from L-lactate that dissolves in H_2_O and CO_2_. PLLA was used as a material for human orthopedic implants [15] and as synthetic material in ligament or tendon tissue engineering research [16]. An *in vitro *study that compared PGA, poly-L-lactate-epsilon-caprolactone (PLC), and PLLA scaffolds concluded that PLLA and PLC scaffolds provided the best material for tendon regeneration because of slow absorption rates and better mechanical properties [17]. Augmentation of rotator cuff repair with a PLLA scaffold provided sufficient initial strength in a sheep model [16], but the byproducts of PLLA degradation might have inhibited the tendon from healing to bone [18]. In present study, the ultimate failure load of the scaffold group increased over time and was not different from normal infraspinatus tendon 8 weeks after surgery. No difference was observed between the scaffold group and reattachment control group, indicating that the implantation of the layered PLLA scaffold in rotator cuff defect had same strength as reattachment. Thus the authors could confirm our hypothesis that PLLA scaffold without cultivated cells would lead to regeneration of tissue with mechanical properties similar to reattached infraspinatus without tendon defects. However, the stiffness was weaker in the scaffold group than normal infraspinatus tendon, indicating that the regenerated tissue had not matured as a normal tendon. While type I collagen is prominent in the normal rotator cuff tendon, type III collagen is expressed during the tendon healing process and its levels decrease as the tendon matures [19,20]. Therefore, if the regenerated tissues mainly consist of type III collagen, the mechanical property of these tissues is presumably inferior to the native tissues [21-23]. Dominant expression of type III collagen was observed, which may have been caused by the low absorption rate of the PLLA fiber that can lead to inferior stiffness properties compared with normal infraspinatus tendon.

There were some limitations in this study. Firstly, the anatomy of a rabbit's shoulder differs from the human rotator cuff because the short rotator muscles of rabbits do not form a rotator cuff as in humans. Secondly, the rabbit model in present study differs from chronic rotator cuff injury model. Thirdly, the PLLA scaffold can induce an inflammatory reaction. Clinical studies showed that a systemic or local reaction might arise. Although the degradation products of PLLA are natural metabolites, they are acidic [14,24]. The absorption rate of the scaffold must be taken into consideration and long-term follow up is required to confirm results.

## Conclusions

A novel form of layered PLLA scaffold was used to repair rotator cuff defect in a rabbit model. This layered form of the PLLA scaffold has the potential to induce cell migration and to fill the gap between the bone and the tendon stump, with mechanical properties similar to reattached infraspinatus.

## Competing interests

The authors declare that they have no competing interests.

## Authors' contributions

TK, HF and MK conceived the study, and participated in its design and coordination. AI performed the statistical analysis and drafted the manuscript. IN, RS, and HN performed the animal experiment and mechanical evaluation. MK and TN provided the PLLA fabric. All authors read and approved the final manuscript.
